# MR-Guided Microwave Ablation for Lung Malignant Tumor: A Single Center Prospective Study

**DOI:** 10.3389/fonc.2022.856340

**Published:** 2022-04-27

**Authors:** Ruixiang Lin, Yan Fang, Jin Chen, QingFeng Lin, Jian Chen, Yuan Yan, Jie Chen, Zhengyu Lin

**Affiliations:** ^1^ Department of Interventional Radiology, First Affiliated Hospital of Fujian Medical University, Fuzhou, China; ^2^ Nursing Department, First Affiliated Hospital of Fujian Medical University, Fuzhou, China; ^3^ Department of Interventional Radiology, Sanming Second Hospital, Sanming, China

**Keywords:** magnetic resonance imaging, lung neoplasms, microwave, ablation techniques, pneumothorax

## Abstract

**Objectives:**

To prospectively investigate the feasibility and efficacy of MRI-guided MWA for lung malignant tumor in our single center.

**Materials and Methods:**

22 patients [mean age, 56.86 ± 13.05(23–73)years] with 23 malignant lung tumors were enrolled in the study. 21 patients had a single lesion and 1 patient had 2 lesions in the ipsilateral lung. The average maximum diameter of the lesion was 1.26 ± 0.65 (0.50-2.58)cm. Percutaneous MWA was guided by 1.5T MRI scanner using a MR-compatible microwave antenna to the target the lung lesions and ablation area was monitored intraoperatively by using a shielded MR-compatible microwave device and then follow-up.

**Results:**

All patients were successfully treated under MR-guided MWA for lung tumors. Average operation time was 72.21 ± 24.99 (36–158) mins. T2WI signal intensity of the lesion gradually decreased over the course of MWA. The center of the ablated zones showed a short T1 and short T2 signals with the ring-like of long T1 and long T2 signals surrounded after immediately evaluation. No serious complications occurred. The average follow-up period was 12.89 ± 4.33 (2.0-19.6) months. Local recurrence occurred in one patient, representing a technical efficacy of 95.5% (21/22).

**Conclusion:**

Magnetic resonance-guided microwave ablation for lung malignant tumor was feasible and demonstrated unique advantages in efficacy evaluation.

## Introduction

Primary lung cancer has the second highest incidence and highest mortality among all cancers, while lung also ranks the second most likely site of tumor metastasis (1). Although surgical resection is effective in treating lung cancer, only about 20% of lung lesions can be surgically removed due to various reasons, making effective alternative therapies for local treatment compulsory (2). Microwave ablation (MWA) is considered as an alternative treatment option for primary and metastatic lung cancers. Owing to its slight trauma and reliable efficacy, MWA has been widely used in cancer treatment in recent years (3, 4). As lung tissue contains air, MWA is predominantly guided by computed tomography (CT) (5). As an alternative approach, magnetic resonance imaging (MRI) has the advantages such as free of ionizing radiation, scanning images in any orientation, sensitive to changes in moisture and temperature caused by MWA. Therefore, it has already been applied to guide tumor ablation in liver, kidney, bone, and soft tissues (6–8). The purpose of this study was to prospectively investigate the technical feasibility and short-term efficacy of MR-guided MWA for malignant lung tumor in our single center.

## Materials and Methods

### Materials

#### Clinical Information

This prospective study was approved by the ethics committee of the institution, and all participating patients signed an informed consent. The inclusion criteria was as follows: (1) No presence of lesions other than the target(s); (2) Diameter of the solitary lung malignant lesion(0.5cm–3cm); (3) Distance between the lesion and hilar vessels or large bronchi >1 cm; (4) No presence of severe emphysema or pulmonary fibrosis; (5) Platelet count ≥50 x 10^9^/L and prothrombin activity ≥40%; (6) ECOG score ≤2; and (7) No contraindications for MRI.

We prospectively analyzed 22 patients with 23 lesions who underwent MR-guided MWA for malignant lung tumors in our center between Jan 2020 and June 2020. Patients’ general information is listed in **Table 1**. Of these cases, 9 were men and 13 were women, with an average age of 56.86 ± 13.05 (23–73) years. Ten cases had primary lung adenocarcinoma, and the remaining 12 had lung metastasis (4 cases of colorectal cancer metastasis, 2 cases of hepatocellular carcinoma metastasis, 2cases of lung cancer metastasis, and 1 case each of gastric, breast, tongue, and giant cell tumor of bone cancer metastasis). In addition, 21 cases only had a single lesion, while 1 case had 2 lesions on the ipsilateral lung. The pathological diagnoses of all cases were confirmed by needle biopsy. The average maximum diameter of the lesion was 1.26 ± 0.65 (0.50-2.58) cm. 11 lesions were located in the upper right lobe, 1 in the middle right lobe, 5 in the lower right lobe, 4 in the upper left lobe, and 2 in the lower left lobe.

**Table 1:** Patient characteristics.

Characteristic	Value
Age	
Average age (years)	56.86 ± 13.05 (23-73)
Gender	
Male	9 (40.91%)
Female	13 (59.09%)
Pathological diagnosis	
Adenocarcinoma	10 (45.45%)
Lung metastasis	12 (54.55%)
Number of lesions	
Single leison	21
Two leisons	1
Location	
Left lung	6 (27.27%)
Right lung	16 (72.73%)
Lesion size (cm)	1.26 ± 0.65 (0.50-2.58)
Operation time (min)	71.77 ± 25.71 (36-158)
Ablation power (W)	81.32 ± 7.31 (60-90)
Ablation duration (min)	5.57 ± 1.71 (3.0-9.0)
Follow-up period (m)	12.89 ± 4.33 (2.0-19.6)

#### MRI

A 1.5T dual-gradient MRI scanner (GE Signa Infinity Twinspeed, USA) and a Torso coil with a rectangular operating hole were used in the study. Scan sequences and parameters were as follows:

1. Fat-suppressed fast-recovery fast spin echo (fs FRFSE) T2WI with repetition time (TR) of 8500.0 ms, echo time (TE) of 85.0 ms, 90°C flip angle (FA), 17 echo train length (ETL), 5.0 mm slice thickness (ST), 1.0 mm GAP, 280 × 380 field of view (FOV), 1 excitation (NEX), and 70 - 90 s scanning time (T).
2. Three-dimensional dynamic T1 weighted imaging (3D Dyn T1WI) with 4.8 ms TR, 1.1 ms TE, 45°C FA, 1 ETL, 3.0 mm ST, 280 × 380 FOV, 1 NEX, and 12 s T.

The 3D Dyn T1WI scan was acquired under breath-hold, whereas the fsFRFSE T2WI scan was acquired with respiratory gating.

#### MWA Device

A MR-compatible MWA device (MTC-3C, 2450 MHz, Vison Medical Co., Nanjing, China) was used in the study. Magnetic shielding was achieved by shielding the host with non-magnetic aluminum alloy plates and placing it outside the 0.5-mT line. In addition, a 2.5-m coaxial transmission cable equipped with a choke coil was used, so that MRI was not interfered during startup and tumor ablation. Lastly, a MR-compatible microwave antenna (14 G, 15 cm, Vison Medical Co., Nanjing, China) was adopted, which was made of copper alloy and ceramics and had a built-in water-cooling cycle.

### Methods

#### Preoperative Preparation

One week before procedure, chest CT scans and chest MRI scans with contrast enhancement were performed to identify the lesion and surrounding tissues. Four hours before procedure, patients were instructed to fasting, and an intravenous indwelling needle was introduced. Subsequently, based on the needs of puncture, patients were placed either in the supine or the prone position, and were connected to the respiratory gating system, underwent training for their breathing patterns, and a skin marker was applied (cod liver oil matrix).

#### MWA Operation

FsFRFSE T2WI and 3D Dyn T1WI scans were first acquired to determine the puncture path and the puncture point on the skin. The puncture path was selected to pass through some normal lung tissue (≥ 2 cm) without touching important structures such as large blood vessels, heart, mediastinum and hilus. Before puncture, routine disinfection was performed and local anesthesia was induced with 2% lidocaine, followed by application of a sterilized hole towel to the puncture point and a sterilized cover for the scanning coil.

A small incision was made at the puncture point by a surgical knife, through which the microwave antenna was inserted into the chest wall and gradually loaded under the guidance of MRI using the fsFRFSE T2WI sequence. During the procedure, efforts were made for the antenna to penetrate the center of the lesion so that its tip surpassed 0.5–1 cm beyond the tumor. Subsequently, once the microwave antenna was connected to the host using the coaxial cable, and the bed was moved to the scanning position, the ablation power and duration were set, the water-cooling cycle was initiated, and the air in the antenna was exhausted. MWA then started, during which fsFRFSE T2WI scans were continuously acquired to monitor the process. Ablation was terminated when its range covered at least 0.5–1.0 cm beyond the tumor. For incomplete ablations, the position of the microwave antenna was adjusted for supplemental ablation. Once the ablation was completed and the indwelling needle was removed, fsFRFSE T2WI and 3D Dyn T1WI scans of both lungs were acquired to evaluate the technical success rate and find any signs of complications. Last, chest radiographs were acquired one day after MWA, and chest CT scans were conducted three days after MWA to rule out perioperative complications.

## Complication

Complications were evaluated based on the guidelines of the Society of Interventional Radiology. The description of complications follows the proposed standardization of terminology and reporting criteria in this study (9).

### Follow-Up and Efficacy Evaluation

One month after MWA, chest MRI with contrast enhancement were performed to evaluate the technical efficacy (9). Subsequent follow-ups were conducted at an interval of 3–4 months using chest CT scans.

### Statistical analysis

Parameters that were recorded included the operation time, power, and duration of MWA, incidence of complications, technical success rate, and technical efficacy. Statistical analysis was performed in the SPSS20.0 statistical software. Measurement data was expressed as average ± standard deviation.

## Results

### MWA Results

All patients were successfully completed MR-guided MWA for lung malignant tumor with a technical success rate of 100%. The average operation time was 71.77 ± 25.71 (36-158) mins. Of all 23 lesions, 12 lesions were treated with single-site ablation and 11 lesions with 2-sites ablation, respectively. The average ablation power and duration were 81.32 ± 7.31 (60-90)W and 5.57 ± 1.71(3.0-9.0) min, respectively. The average maximum ablation diameter was 3.61 ± 0.52cm(2.59-4.49cm) with the minimum ablation diameter of 2.67 ± 0.45cm(1.51-3.39cm), respectively.

### Incidence of Complications

There were 3 cases of pneumothorax either during or after the ablation, with 1 case requiring management by catheterization. One patient had a small amount of hemorrhage during the procedure which stopped after ablation. Ten patients with mostly near pleural tumors experienced intraoperative and postoperative pain, which was alleviated after symptomatic treatment with analgesia. Two patients suffered from a small amount of pleural effusion without catheterization. Other complications included infection (2 patients), which was treated with antibiotics, and low-grade fever (4 patients) which was treated by physical cooling. No serious complications such as bronchopleural fistula and massive hemoptysis were observed in the study.

### Follow-Up Results

The average follow-up period was 12.89 ± 4.33 (2.0-19.6) months. During the follow-up, one case colon cancer lung metastasis of local recurrences occurred 4 months after MWA. The resultant technical efficacy was 95.5% (21/22).

### Intraoperative MRI Manifestations

**Figure 1:**
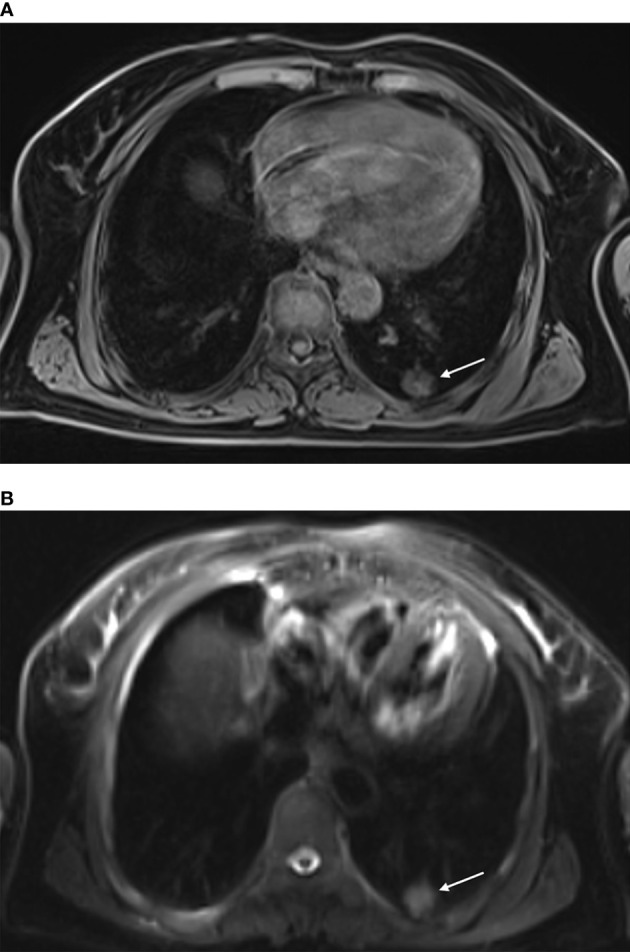
MR-guided MWA for invasive adenocarcinoma of the left lower lobe for 72-year-old woman. Preoperative scan: Preoperative MRI showed a nodule in the left lower lobe, displayed as isointense signals on T1WI [**(A)** arrow] and high signals on fsFRFSE T2WI [**(B)** arrow].

**Figure 2:**
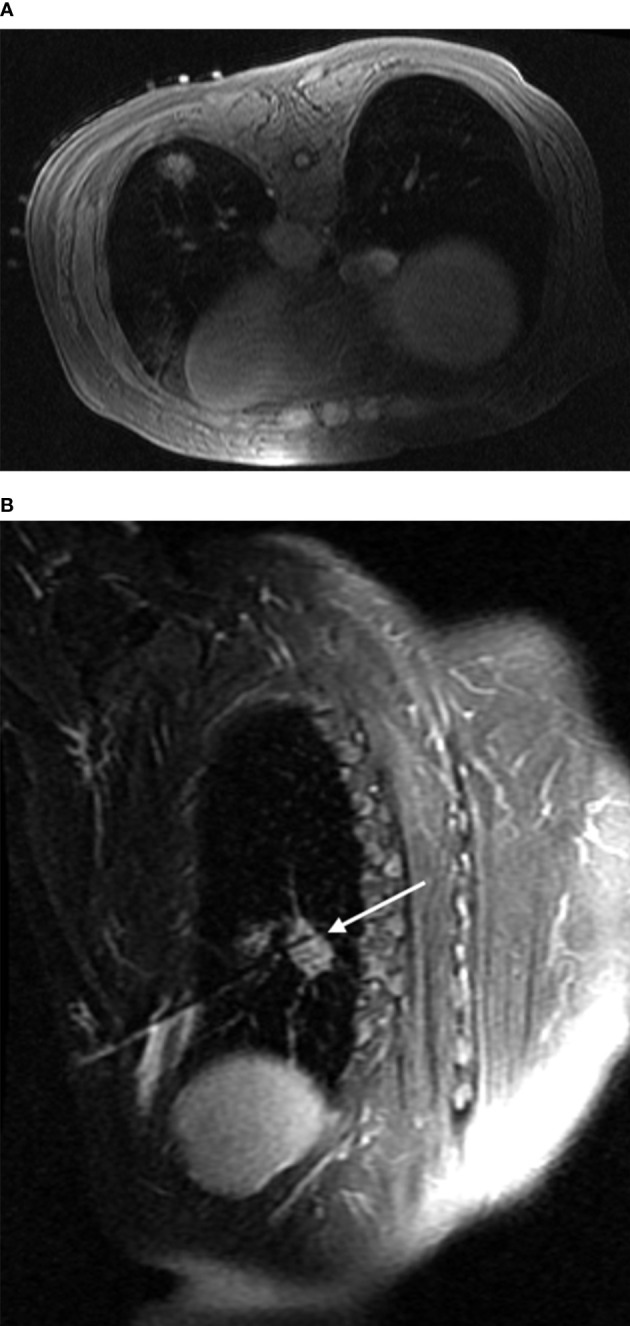
Intraoperative scan: Intraoperative scan was acquired with the patient in the prone position. The lesion showed isointense signals on T1WI, and the hyperintensity marker (cod liver oil matrix) was visible on the patient’s surface **(A)**. In the oblique sagittal fsFRFSE T2WI view with the microwave antenna as the long axis, the lesion was seen as high signals, while the microwave antenna displayed as a strip of low signals surrounded by a small amount of high signal due to hemorrhage [**(B)** arrow].

**Figure 3:**
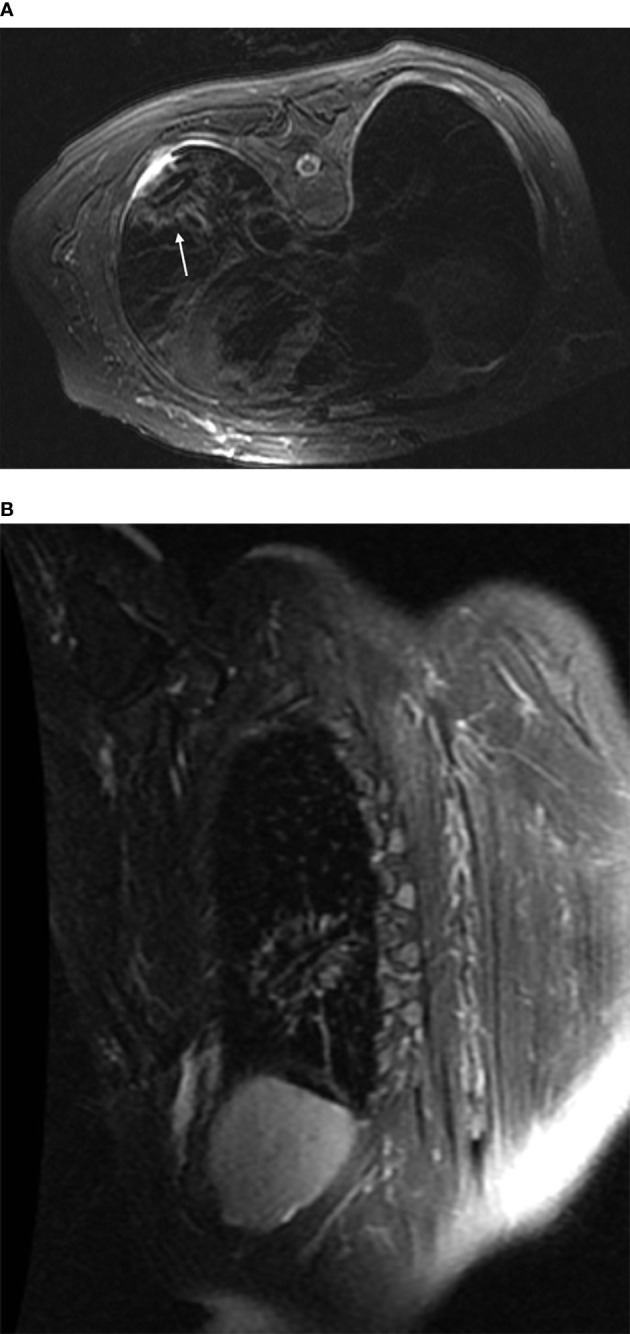
Post-ablation scan: In both the transverse **(A)** and the oblique sagittal **(B)** views fsFRFSE T2WI scans, the ablation foci displayed as obvious low signals surrounded by ring-shaped high-signal shadows. In addition, the microwave antenna displayed as a strip of low signals, and a small amount of fluid was seen in the pleural cavity adjacent to the lesion.

Compared with the chest wall muscle, prior to ablation, lesions showed isointense or slightly low signals on T1WI images (**Figure 1A**). Alternatively, on T2WI images, all lesions showed high or slightly high signals (**Figure 1B**). During the intraoperative scan (**Figures 2A, B**), T2WI signal intensity of dynamic scan showed the zone of coagulative necrosis area with hypointense gradually expanded from the center to the outer periphery and covered the hyperintense lesion within time advances. Last, during the postoperative scan, tumors showed decreased T2WI signals surrounded by high signals (**Figures 3A, B**) and increased T1WI signals surrounded by isointense signal bands.

## Discussion

### Microwave Ablation Guidance

The large amount of air in the lungs provides a natural contrast on computed tomography images. In addition, computed tomography can accurately evaluate ablation complications such as pneumothorax as well as clearly displaying the metal ablation antenna. Therefore, despite its ionization hazard, computed tomography remains the primary guidance method of microwave ablation for lung cancer, positron emission tomography-computed tomography and C-arm computed tomography are based on the same principles (10–12). Microwave ablation for lung cancer is considered successful if the ground glass opacity on the immediate post-ablation computed tomography covers 0.5–1.0 cm beyond the lesion (13). However, the boundary of the ground glass opacity is often unclear on computed tomography and can be covered by pulmonary hemorrhage as a result of puncture, and unobvious density changes in ablated tumor (14).

On the contrary,MR guided MWA has unique advantage including non-radiation especially at young patients, high solution of soft tissue, more precise efficacy assessment and can measure temperature changes non-invasively (14). In this prospective study, we select MR-guided for diameter of lung malignant lesion beyond 5mm and solitary nodule. Therefore, this study investigated the use of MRI to guide MWA for malignant lung tumors.

### Feasibility of MR Guidance

#### Devices and Instruments

MR-guided MWA requires devices and instruments not only made of non-ferromagnetic, but also electromagnetic compatible materials, so that MRI can be acquired simultaneously during ablation without mutual interference. The MWA device and the microwave antenna adopted in this study were both made of non-ferromagnetic materials, allowing them to be placed in the MR scanning room. As a result, connecting cables did not need to be extended, thereby avoiding a substantially reduced ablation range due to microwave output loss. In addition, the MWA device was electromagnetically shielded, while the connecting cable was equipped with a choke coil according to the frequency of the MRI scanner’s primary magnetic field (63.637 MHz). Consequently, ablation did not disrupt concurrent MRI, thereby making it possible to perform real-time monitoring of the ablation process to prevent excessive or insufficient ablation.

#### Microwave Antenna Display

Although the microwave antenna displays clearly in the soft tissue of the chest wall, once it enters the lung tissue with air, neither the antenna itself nor the lung tissue produces a signal on the MRI. Therefore, the fsFRFSE T2WI sequence was adopted in this study to guide the puncture, so that high signals generated by different degrees of pulmonary hemorrhage during puncture could be used to project the non-signal microwave antenna. It was possible to accurately guide the puncture process by comparing the *in vivo* depth of the microwave antenna with the depth measured on the MRI.

#### Efficacy and Complications

Among the 22 patients who underwent MR-guided MWA in this study, the technical success rate (100%), the technical efficacy (95.5%), and the incidence of complications were not inferior to those of CT-guided MWA (15–18). MR was also capable of indicating complications such as pneumothorax and hemorrhage and demonstrated unique advantages in efficacy evaluation. Studies have shown that MRI manifestations has a strong correlation with pathological findings (19, 20). In a study performing MWA on rabbit lung VX2 tumors, Chen Jian et al. found that lung VX2 tumors with high signals on T2WI and isointense or low signals on T1WI before MWA, showed significantly reduced T2WI signals and increased T1WI signals after MWA due to coagulation necrosis and dehydration. In contrary, due to inflammatory reaction after thermal injury, the surrounding lung tissue experienced an increase in water content. This led to high signals on T2WI and isointense signals on T1WI that formed a clear contrast with the ablation foci, thereby facilitating a more accurate identification of the ablation boundary. Meanwhile, Chen Jin et al. (14) performed MR scans on 20 patients with lung cancer immediately after CT-guided radiofrequency ablation and found that MRI was accurate and reliable in the efficacy evaluation. In addition, they reported that bleeding during the puncture process displayed as low signals on T1WI, resulting in significant signal differences with the high signal of ablation foci and the isointense signal of thermal injuries that allowed MRI to provide a better efficacy evaluation than CT. In our study, as the study mention above, the center of the ablated zones showed a short T1 and short T2 signals with the ring-like of long T1 and long T2 signals surrounded by beyond 5mm after immediately evaluation considered as complete ablation.

This study has some shortcomings. First, only 22 cases were included in the study, most of them had a short follow-up period with small lesions, making studies with a larger sample size and a longer follow-up duration necessary. Second, after puncture into the lung, the display of the microwave antenna tract on MRI was not as clear as that on CT, causing a substantially longer operation time of the former. Third, due to the vascular flow effect of MRI, we could not determine the incidence of air embolism, a rare but serious complication.

In conclusion, magnetic resonance-guided microwave ablation for lung malignant tumor is feasible and has unique advantages in efficacy evaluation, making it suitable for guiding microwave ablation for lung lesions in the future.

## Data Availability Statement

The raw data supporting the conclusions of this article will be made available by the authors, without undue reservation.

## Ethics Statement

The studies involving human participants were reviewed and approved by the ethics committee of first affiliated hospital of fujian medical univercity. The patients/participants provided their written informed consent to participate in this study

## Author Contributions

ZL conceptualized the study, prepared figures and tables.RL wrote the article and prepared figures and tables. YF collected the data, carried out the analysis, and prepared the figures and tables. JinC, QL and JiaC participated in drafting and editing the article and assisted in the preparation of figures and tables. YY and JieC participated in figure preparation and drafting and editing the article. All authors contributed to the article and approved the submitted version.

## Funding

This study was funded by The key Research and Development Programs of Jiangsu,PRC(Social Development Clinical Frontier Technology)(BE2017758),and The Research and Develop Program of Focus Field of Guangdong Province,PRC (2019B110233001).

## Conflict of Interest

The authors declare that the research was conducted in the absence of any commercial or financial relationships that could be construed as a potential conflict of interest.

## Publisher’s Note

All claims expressed in this article are solely those of the authors and do not necessarily represent those of their affiliated organizations, or those of the publisher, the editors and the reviewers. Any product that may be evaluated in this article, or claim that may be made by its manufacturer, is not guaranteed or endorsed by the publisher.
